# Nightmares in Migraine: A Focused Review

**DOI:** 10.3390/bs11090122

**Published:** 2021-09-04

**Authors:** Parisa Gazerani

**Affiliations:** 1 Department of Life Sciences and Health, Faculty of Health Sciences, Oslo Metropolitan University, 0130 Oslo, Norway; parisaga@oslomet.no or gazerani@hst.aau.dk; 2 Department of Health Science and Technology, Faculty of Medicine, Aalborg University, 9220 Aalborg E, Denmark

**Keywords:** nightmares, pain, sleep, sleep disorders, migraine, headache

## Abstract

Nightmares usually occur during the sleep phase of rapid eye movement (REM) and are associated with some physical symptoms, including sweating, shortness of breath, and lower limb movements. Emotions of fear, anger, shame, and sadness may also accompany nightmares. These symptoms can occur during dreaming, upon awakening, or later when the dream experience is recollected. Nightmares may sporadically occur for everyone, but nightmare disorders are associated with features of impaired mental and physical health and require professional medical treatment. The occurrence of nightmares with several disorders has been reported in the literature, but in migraines it has only been investigated in a small number of studies. Considering the existing relationship between sleep disorders and migraine, the occurrence of nightmares in migraine can negatively affect this association and elevate the risk of depression and anxiety. This, in turn, further reduces the quality of life of affected individuals. Hence, expanding the knowledge on the link between nightmares and migraine, promoting an acceptable quantity and quality of sleep through pharmacological and nonpharmacological interventions in the management of nightmares in migraine, and further scientific investigation of the biopsychosocial mechanisms underlying the link, will be highly valuable for optimal care. This focused review, therefore, gives a brief overview of the current understanding of nightmares in migraine to highlight the open questions and value of further research. The ultimate goal is to contribute to timely recognition and sufficient action to offer beneficial outcomes for affected patients.

## 1. Introduction

### 1.1. Sleep Disorders and Pain

Sleep disorders and pain often coexist and lead to serious negative effects on health and quality of life [1]. Sleep disorders have been registered in about 90% of patients with chronic pain, and half of patients with insomnia complain of pain [2]. A bidirectional relationship between pain and sleep disorders has been proposed [3,4], suggesting that pain can disrupt sleep, and disturbed sleep, in turn, enhances pain [5]. This relationship acts to sustain or amplify sleep deficiency and pain through a vicious cycle [5]. The structure of sleep disorders in patients with chronic pain is similar to that observed for patients with primary insomnia [6]. A positive correlation between the intensity of chronic pain and the degree of sleep disturbance has also been reported [7]. Even though evidence exists of this reciprocal relationship, the neurochemical underlying mechanisms remain less investigated [5].

### 1.2. Sleep Disorders and Headaches

Among chronic pain conditions, chronic headaches are highly prevalent comorbid conditions with sleep disorders [8,9]. An earlier large epidemiological study [10] showed that 18.1% of the studied population had concurrent headache and insomnia, 16.3% only had headaches, and 21.1% only had sleep disturbances. The comorbid condition was prevalent among women and middle-aged individuals and the risk factors identified were a low socioeconomic level, unhealthy lifestyle, stress, anxiety, and depression [10]. Based on these findings, timely diagnosis, treatment, and prevention seem essential. For example, preventive strategies such as lifestyle modification, reducing stress and its impact, and properly dealing with depression and anxiety may offer great value for the prevention of headaches and sleep disorders [10].

The comorbidity between headaches and sleep disorders has been explained by shared neuroanatomical structures within the nervous system and neurobiological and psychological factors involved in both headaches and sleep disorders [8,11,12,13,14].

**Figure 1:**
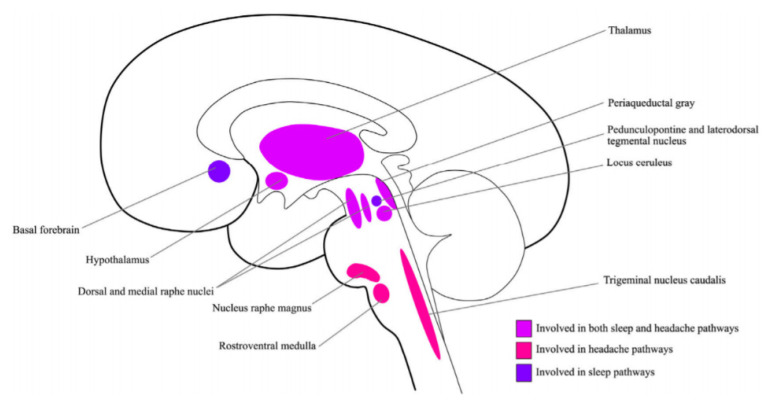
The key structures involved in sleep and headache pathways [19] (reused with permission, license number: 5132930606535, Elsevier and Copyright Clearance Center).

From an anatomical point of view, there are structures where pathways of sleep and headache cross [15], including the thalamus, hypothalamus, locus coeruleus, and periaqueductal gray [8,16,17,18]. Figure 1 presents the structures that are proposed to underlie comorbid conditions of sleep disorders and headaches [19]. Therefore, studying how these structures function [20] when headaches and sleep disorders coexist can help us to understand the underlying mechanisms.

From a biochemical point of view, several neurotransmitters and neuromodulators have been proposed that most likely play important roles in both sleep disorders and the pathophysiology of headaches. Among them, adenosine [5,21,22,23,24,25], dopamine [2,11,26], melatonin [27,28,29,30,31,32,33,34,35], orexin [9,15,16,18,36,37,38], and serotonin [39,40,41,42,43] are proposed as key elements.

Psychological factors are also known to play a role in the relationship between headaches and sleep disorders [8]. These factors can lead to anxiety [44,45,46], depression, and a poor quality of life [10]. For example, to explain the link between chronic insomnia and chronic headaches, a bio-behavioral model [47] has been proposed. In this model, chronic headache enforces maladaptive behaviors such as excessive time spent in bed, use of sleeping aids before sleep, and consuming stimulating drinks during the day [48,49] that collectively create a vicious circle and worsen sleep. This, in turn, leads to dramatic and catastrophizing attitudes towards head pain [50]. Therefore, paying attention to sleep history when interviewing patients with headaches can be beneficial for correcting maladaptive behavioral strategies and breaking the vicious circle [13].

The establishment of a causal relationship between sleep disorders and chronic headaches, i.e., “which one comes first?”, is challenging in patients who suffer from both conditions; however, a consensus exists to support the notion that the comorbidity of sleep disorders and headaches enhances the risk of chronification for both conditions and increases the burden of both disorders, with a higher frequency of complications and lower quality of life [2]. For example, patients with insomnia have a 2- to 3-fold higher risk of headaches of different types, including migraine, tension-type headaches, and chronic daily headaches [51]. The severity of sleep disorders also is correlated with increased headache frequency [52]. Sleep seems to play a multidimensional role in relation to headaches and acts as a headache reliever (i.e., sleep can often abort headaches, if patients manage to fall asleep), a headache provocative factor (i.e., sleep deprivation can trigger headache attack), and a headache modifier (i.e., sleep disorders, such as sleep apnea can lead to secondary headaches and alter manifestation of primary headaches) [8,53]. In this regard, several recent reviews and meta-analyses are available [8,9,13,38] demonstrating the relationship between headaches and sleep disorders that highlight the importance of this complex interaction and open challenges/possibilities of effective therapeutic strategies.

### 1.3. Sleep Disorders and Migraines

Sleep disorders and migraines are prevalent and pose a high health and socioeconomic burden [13]. Similar to chronic headaches in general, a complex relationship between migraine and sleep disorders has been reported [13,14], and although the exact underlying mechanisms are not known [11], shared anatomical structures (see Figure 1) and neurobiological mechanisms have been proposed to underlie the link [13,14,54]. A likelihood of different psychological factors has also been presented. Table 1 summarizes the major neurotransmitters/neuromodulators proposed in the literature [5,13,14,22,55,56] as potential mechanisms underlying the sleep disorder and migraine comorbid condition. Please note that a number of other neurotransmitters, neuropeptides, neuromodulators, hormones, and proinflammatory substances are also proposed to contribute to the link between sleep disorders and pain, but hypothesis-driven qualified data are needed to support the link between sleep disorders and migraine in particular.

Both quality and quantity of sleep [57] are valuable factors to take into account when dealing with the sleep–migraine link. For example, the duration of sleep does not seem to be different between migraine and non-migraine patients [58,59]. However, poor sleep quality is shown to be linked to higher headache frequency [60], depression, and anxiety [61]. Excellent reviews are available of migraine and sleep disorders and readers are referred to them [8,11,13].

### 1.4. Nightmares among Sleep Disorders

Many types of sleep disorders, such as insomnia, sleep apnea, restless leg syndrome, and narcolepsy, are often seen among the general population [18]. Within the context of sleep disorders, nightmare disorders [17] have been recognized that can manifest with features of impaired mental and physical health. Nightmare disorder is relatively rare and is characterized by frequent occurring of nightmares that cause distress, disrupt sleep, cause problems with daytime functioning, or create fear of going to sleep. Nightmare disorder is defined in the Diagnostic and Statistical Manual of Mental Disorders, Fifth Edition (DSM-5) as “*repeated awakenings with recollection of terrifying dreams, usually involving threats to survival, safety or physical integrity*”. The International Classification of Sleep Disorders, third edition (ICSD-3) [62], defines nightmare disorder as “*a parasomnia (i.e., abnormal or unusual nervous system behavior during sleep) usually associated with rapid-eye-movement sleep*”. Recurrent awakening from disturbing dreams and alertness on awakening, accompanying a clear recall of dreams, are among the criteria [17]. If nightmares appear as nightmare disorders, proper diagnosis and treatment are required.

Nightmares usually occur during the REM sleep (the phase with rapid eye movement) and are associated with physical and emotional symptoms. Sweating, shortness of breath, and lower limb movements are common physical symptoms, while fear, anger, shame, and sadness are among the widely reported emotional features. These can occur during a dream, upon awakening, or later when the dream is recollected. Nightmare affects around 5% of adults in the general population [63,64]. For psychiatric population (e.g., patients with posttraumatic stress disorder (PTSD) [65], patients with borderline personality disorder [66], and patients with schizophrenia [67]), however, this estimation appears higher.

Nightmare occurs more often among young individuals and is more prevalent among females [68]. Life stressors and broadly negative affect have been found to precipitate the occurrence of nightmares [63,64]. Evidence supports that a stressful event (e.g., stress related to an exam, natural disasters, stress, and grief related to death of significant other) can lead to more frequent nightmares [64]. Nightmares also are found associated with self-harm and suicidal behavior [69].

Two main types of nightmares have been observed [68]. Post-traumatic nightmares [17,68,70] are manifested as a replication of a traumatic event or a trauma-related emotion. Idiopathic nightmares [71], on the other hand, are more imaginative and might be free from a traumatic event. Strong arousal, nocturnal awakenings, aggressiveness, and helplessness are more often seen with post-traumatic nightmares when compared to idiopathic nightmares [17,68]. Advanced neurophysiological and psychological studies can help to determine the risk factors and underlying mechanisms of nightmares and how to optimally target them [68,72,73].

In addition to psychiatric disorders, nightmares co-occur with several other disorders. Our knowledge is limited about nightmares and chronic headaches, and in migraine, large studies with proper designs are needed to determine the prevalence and characteristics of this comorbid condition. Sporadic evidence exists to demonstrate that nightmares occur in migraine. Due to the limited number of available studies, however, a systematic review of nightmares in migraine is not feasible, and no meta-analysis can consequently be performed at present.

Here, in this focused review, a brief overview is presented of the current understanding of nightmares in migraine. The purpose is to highlight the value of expanding the knowledge on the link between nightmares and migraine, promote an acceptable quantity and quality of sleep through pharmacological and nonpharmacological interventions in the management of nightmares in migraine, and motivate further scientific investigation of the biopsychosocial mechanisms underlying the link. The ultimate goal is to highlight the open questions and value of further research to contribute to timely recognition and sufficient action to offer beneficial outcomes for affected patients.

## 2. Nightmares and Migraine

Among the various types of sleep disorders studied and presented in migraines, the occurrence of nightmares in migraines has been reported sporadically but less often investigated or published about. One study [74] presented persistent nightmares of childhood onset returning in those with migraine compared with those without. The association of nightmares with migraine is independent of lifetime or current anxiety or mood disorders. There are a few studies that have mentioned nightmares linked to anxiety and mood disorders and comorbidity with migraine; however, these links remain underdeveloped. It has been found that individuals who have unpleasant nightmares experience more nocturnal migraine attacks [75,76]. The initial theory was that a persistent negative affective drive in a dream can activate physiological responses that can lead to migraine attacks [75].

### 2.1. Dreams, Unpleasant Dreams, Nightmares, and Migraine

According to Freud, dreaming is a manifestation of the unconscious that reflects the emotional reaction of an individual to his or her surroundings [77]. Dreams have also been defined as the medium for the communication of relevant emotions that occur between a dreamer and the self and others in the surrounding environment [77]. Dreams and emotions are indeed related [78,79,80,81]. The neurocognitive theory of dreams [82] considers that dreams are generated by complex forebrain mechanisms that are independent of the state of REM. This theory is based on neuropsychological findings, where the mesocortical–mesolimbic dopamine system was found to play a role in dream generation. The etiology of nightmare disorder has also been explained by increased hyperarousal that accumulates during the day and is maintained at night. Normal sleep may enable fear extinction, but this system seems impaired in individuals with nightmare disorder and continues to activate, arousing memory during sleep and reinforcing fear.

Dreams in attacks of migraine were investigated by Harold Levitan [75]. He divided the collected dreams into six categories based on their content of manifestation: (1) a dream predominantly containing feelings of terror, (2) a dream predominantly containing feelings of frustration, (3) a dream predominantly containing feelings of loss, (4) a dream predominantly containing feelings of pleasure, (5) a dream containing scenes of incest, and (6) a dream containing oversized creatures. Dreams of terror constitute the largest category. Only one category was not associated with a negative affective aspect, where a positive dream experience preceded a migraine attack (dreams of pleasure). Interestingly, most of the situations that preceded migraine attacks in dreams were found to be more intense than those situations that preceded them when the subject was awake. It was postulated that this might be a result of a lower threshold for the precipitation of migraine in wakefulness compared with that following dreams or the complexity of daily life, which might affect the responsiveness of individuals differently from sleep conditions. Interestingly, it appeared that, rather than a single bad dream, a repeated pattern causes migraine attacks, which is also supported by other findings [76].

Nightmares can provoke attacks in both asthma and migraine. Those pre-attack nightmares were compared by Levitan [77]. The pre-migraine dreams were compared with the pre-asthmatic dreams studied in another study and found to be similar. A total of 61% of the pre-migraine dreams and 43% of the pre-asthmatic dreams were of the type where dreamers were the victim of an aggressive act. However, in dreams where dreamers were the perpetrators of an aggressive act, 27% of the asthmatic dreams were of this type, but there was no indication in migraine patients. Therefore, dreams that can exacerbate or trigger attacks in different conditions might overlap and appear specific. It is not known yet why the active expression of aggression in dreams might contribute to asthma attacks but not migraine attacks.

#### 2.1.1. Dreams as Diagnostic Tools

Lippman, in 1954, considered dreams as diagnostic tools for migraines [76]. He indicated that three different dream patterns were most common, and recognition of those dreams can contribute to the diagnosis. He described similar feelings of horror or terror in the dreams associated with migraines. He pointed to the presence of size-change phenomena in *Alice in Wonderland*. In contrast to the types of dreams discussed earlier, which appear to precipitate the migraine attack, dreams containing size-change phenomena indicate that the migraine attack is already present.

#### 2.1.2. Dreams as Provoking Factor of Nocturnal Migraine Attacks

Heather-Greener et al. [77] studied whether dreams and nocturnal migraine are linked. Hall and Van De Castle’s classification [83] was used in this study to categorize the contents of dreams, and five variables were selected as follows: aggressive interactions, failure in problem solving or a task, misfortune with no control, anger, and apprehension. In total, 37 migraineurs were included in this study [77]; they recorded 10 dreams, of which 5 led to a migraine attack. The findings showed that anger, misfortune, apprehension, and aggressive interactions could predict nocturnal attacks of migraine [77].

It has been known that nocturnal migraines can be triggered or worsened if an intense emotion is experienced or suppressed during the day or sleep [75]. Therefore, resolving issues can help affected patients. According to the literature, patients with migraine seem to suppress and repress aggression quite often while they are awake [84]. Somehow, in these patients, an inability to express emotional conflict and anger [84,85] has also been reported. Evidence exists to support the idea that emotional suppression is linked to somatic symptoms—for example, those seen in migraine [86]. Generally, the evidence is in favor of an association between the suppression of anger and anxiety and unpleasant dreams [80,81,87]. Hence, it is important to guide patients by taking advantage of therapists to overcome their intense emotions, such as anger.

#### 2.1.3. Personality Traits, Nightmares, and Migraine

Personality traits have also been linked to nightmares [63]. A study in 2010 stated that the prevalence of frequent nightmares (defined as at least once per week) is 5.1% in the general population. Female sex, low income, insomnia, sleep apnea, and sleep-related daytime consequences were significantly associated with nightmare frequency. The risk of having a psychiatric disorder was 5.74 times greater for subjects with frequent nightmares, especially mood disorders. Even with the exclusion of concomitant psychiatric morbidities, subjects with frequent nightmares still scored significantly higher on neuroticism in the personality scale. Prospective studies can investigate how various personality traits among migraineurs can be linked to nightmares—for example, whether the obsessive compulsory trait has been found to be associated with perfectionism and whether this can lead to frustration and anxiety. Job-related stress is another situation that seems to be associated with performance issues; if it is not addressed, anger and frustration develop. This might also affect migraineurs, who might face frustration over lack of performance at their job while suffering a migraine attack. Hence, workplaces must acknowledge and facilitate the optimal performance of these patients. We still do not know if this is sex or gender dependent [88], but further investigation might be required to implement suitable strategies. It has also been noted that migraines are attributed to unconscious childhood conflicts triggered by present stressors [84]. Therefore, taking a history of childhood trauma or abuse may identify whether these factors may reflect the fear, anger, and apprehension associated with the content of unpleasant dreams [77]. It is also well known that some drugs used for the preventive therapy of migraine are associated with unpleasant dreams [89]; for example, cases have reported nightmares as a consequence of tricyclic antidepressants (TCA) or beta-blockers [90].

#### 2.1.4. Content of Dreams in Migraine Compared with Nonmigraine or Other Headache Types

In 2015 [91], a cross-sectional study evaluated the content of dreams in patients with migraine. The categories of dreams were based on the Hall and Van De Castle classification [83], which defines 11 dreaming content categories. The contents of friendliness, sexuality, and bad fortune were dominant in patients with nocturnal attacks. The contents of sadness, bad fortune, aggression, confusion, sexuality, and failure could influence the onset of headache attacks. Based on these results, the authors proposed that both positive and negative emotions seem to exacerbate headaches.

De Angeli et al. [92] investigated migraine patients, patients with tension-type headaches, and healthy individuals to evaluate dream content. Compared with healthy people, patients with migraine reported frequent fear and anguish during dreaming, independent of their anxiety and depression. The authors proposed that peculiar features of dreams in patients with migraine might be due to the negative sensations following recurrent migraine or a reaction of the mesolimbic structures activated in dreams and migraine [92].

Lovati et al. [93] included 219 controls, 148 migraineurs, and 45 tension-type headaches to evaluate dreams in different headaches. They found that migraineurs, particularly migraine with aura, had an increased frequency of taste and olfactory dreams and found that anxiety and mood did not influence these results. Based on the findings, they proposed that increased frequency of taste and olfactory dreams among migraineurs seems to be specific and possibly reflects a particular sensitivity of gustative and olfactory brain structures and points to a potential role of the amygdala and hypothalamus. These structures are known to play a role in migraine, sleep, and dreaming [93]. These researchers proposed that the brains of migraineurs might dream differently. However, no study with a brain imaging technique has provided evidence to confirm or disprove these theories. We still do not know if the specific the dreams seen in this study are part of the dysfunctional state of migraine or are caused by the migraine course.

#### 2.1.5. Miscellaneous

A study in 2013 [94] investigated the frequency of dream-enacting behaviors (DEBs) in migraine and found a higher frequency of DEBs in migraine compared with controls. This study was based on the evidence in the literature about the presence of sleep disorders, nightmares, and visual hallucinations in migraine that collectively points to impaired REM sleep. The researchers found that DEBs were linked to impaired sleep and severe headache-related disability in migraine patients [94], and proposed that brainstem dysfunction and increased brain excitability in migraine patients might generate the condition.

Dream studies in migraine can elucidate how physiological and psychological factors can interact with wakefulness and sleep in migraine. For example, if an emotional reaction can be modulated or can act as a predictive value, it might have an implication for therapeutic intervention. Both pharmacotherapy and behavioral treatment of nightmares seem to help individuals [17,95].

## 3. Interventions to Manage Nightmares in Migraine

Both pharmacological and nonpharmacological interventions have been proposed to help with the prevention or treatment of comorbid conditions of sleep disorders and migraine [8,13]. More widespread use, ease of administration, and limited unwanted side effects or complications of nonpharmacological interventions have made these a starting point for treatments that can be applied as lifelong strategies, if required. For example, the successful use of cognitive behavioral therapy (CBT) and its effectiveness for comorbid conditions of chronic pain and sleep disorders [96] has been expanded to chronic headaches [97], including migraine [53,98,99]. There is ample evidence that psychological approaches to headaches are effective, and a behavioral approach called learning to cope with triggers (LCT) has been effective for chronic headaches [100]. CBT-i, or CBT for insomnia, has been recommended as first-line therapy for insomnia [101], and can increase both REM and non-REM (NREM) sleep [102]. Several narrative reviews [100,103,104] and one systematic review with a meta-analysis [9] exist to demonstrate psychological and behavioral techniques, and combining behavioral techniques with medication is effective for reducing headache frequency and intensity and improving sleep parameters such as sleep quality and sleep time. The neurophysiology of psychological sleep interventions and how they can improve headaches are not fully explored, but evidence exists to demonstrate some neurophysiological changes that occur in the brain regions that are known in both pain and sleep [15]. Subcortical resting-state functional connectivity was studied in patients with insomnia pre- and post-application of CBT-i [105], and a decrease in functional connectivity was found between the thalamus and the parietal cortex [105]. This decrease was inversely correlated with sleep efficiency. The measurement of regional cerebral blood flow [106] showed a mean increase of 19% in blood flow to the thalamus during non-REM sleep when it was measured pre- and post-behavioral therapy for insomnia.

Other behavioral sleep modification strategies include sleep restriction/bed restriction, stimulus control, and sleep hygiene [107]. Sleep hygiene is one of the main components to maintain an optimal lifestyle. Consumption of alcohol at night can influence and fragment sleep and hence should be avoided. Consuming nicotine and caffeine before bedtime can also prohibit proper sleep as these substances are stimulants. Environmental factors such as noise and light must be controlled and are normally monitored and adjusted when sleep hygiene is being consulted.

A case [108] is also presented in the literature with a complete resolution of pain after a lucid dream when a biopsychosocial treatment was applied for two years [108]. A potential central nervous system reorganization known as neural plasticity was therefore proposed to underlie the therapeutic effect of lucid dreams [108].

While most studies have reported the above-mentioned interventions for breaking the vicious cycle of chronic pain and sleep disorders, reports that demonstrate the effect on nightmares and migraine are limited. Hypnosis, desensitization and reprocessing of eye movement, and deep-muscle relaxation have been mentioned for the treatment of nightmares in general [17]. We still do not know whether these techniques can help patients with migraine. A method called behavioral intervention with imagery rehearsal therapy (IRT) seems beneficial for the treatment of nightmares [17]. This method is based on the theory that nightmares are a learned behavior and can be replaced by less disruptive behavior. To apply IRT, affected patients are asked to remember the nightmare, write it down, and change its content to a more positive version that can be practiced as a re-transcript dream [17]. This technique seems feasible and might also help migraine patients to overcome their nightmares.

Some medications can be used for nightmare treatment (for example, prazosin; atypical antipsychotics such as aripiprazole, olanzapine, and risperidone; benzodiazepines such as nitrazepam and triazolam; topiramate; and TCAs). These drugs are often used for nightmares related to PTSD. One may imagine that these can also be effective to limit nightmares in migraine, but it must be kept in mind that some medications can contribute to nightmares. For example, propranolol or TCAs, used as prophylactic agents for chronic migraine, can also contribute to nightmares. Therefore, the choice of drugs for preventive therapy in susceptible patients is important. In general, the dose and timing of pharmacological treatments should be considered when multiple comorbidities are present, such as nightmares and migraine.

Collectively, a specific strategy or guideline to treat the comorbid condition of nightmares in migraine is still needed, but the methods presented above are likely to help. In addition, Gieselmann et al. [68] provided an overview of the etiology and treatment of nightmare disorders, where different treatment options are presented based on the meaning of nightmare dreams, persistence and repetition of nightmares, and maladaptive beliefs. The strategies presented in this paper might also be applicable to nightmares in migraine to some extent.

## 4. Concluding Remarks and Future Perspectives

**Figure 2:**
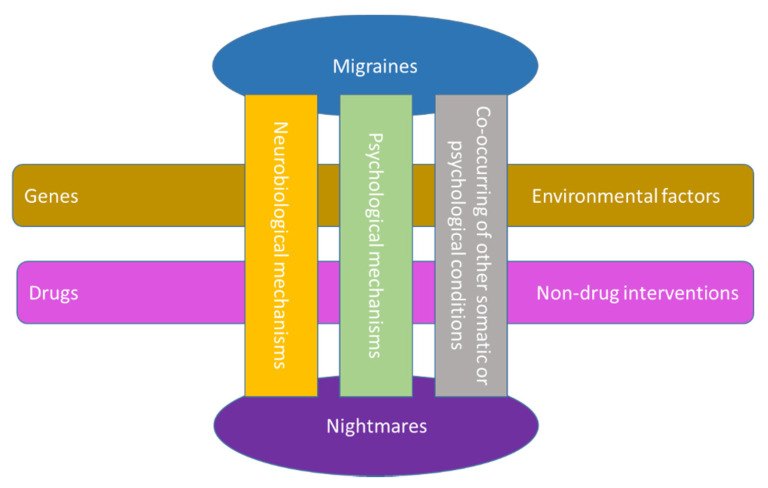
The proposed link between nightmares and migraine, and the potential factors influencing this link.

Chronic headaches are comorbid with several disorders, one of which is sleep disorders. Nightmares and migraine are two distinct entities from both disorder groups (i.e., headaches and sleep disorders). Evidence from the literature demonstrates that nightmares sometimes accompany migraine, and this link has been proposed as a result of number of factors that affect both migraine and nightmares, for example, various stressors. Hence, an investigation of their interaction can help us to identify common neurobiological or psychological pathways and the effects of nonpharmacological interventions and optimal pharmacological agents. Comorbid factors and the roles of environmental and genetic factors can also be studied. Figure 2 depicts the proposed link between nightmares and migraine and the potential factors influencing this link.

Dream studies, combined with imaging techniques and functional and biological assays in migraine under and without interventions, can aid our better understanding and management of nightmares in migraine.

In the clinic, it is important to decide if the nightmare in migraine is a nightmare disorder or not, as proper evaluation and professional treatment might be required. It is also critical to identify exacerbating factors among migraine patients such as sex, age, drug use, personality traits, beliefs, coping strategies, and other comorbid conditions. In addition, the content of a dream can be collected and investigated to identify whether psychiatric disorders are comorbid with migraine. This can assist with the proper and timely selection of an optimal procedure for treatments. Considering the current trend of patient-centered care, the education and active involvement of patients suffering from this co-morbid condition can be highly beneficial. Since disturbing dreams may serve as an alarm signal [109], it is important to carefully evaluate and consider forms of psychopathology and mental disorders while dealing with nightmares in migraine. Besides psychological and behavioral techniques and combining behavioral techniques with medication, lifestyle modification seems to play a critical therapeutic role in both migraine [110] and sleep disorders [111]. For example, dietary factors [112,113] and exercise [114] interact with both migraine and sleep [115] and may influence nightmare incidence in migraine. This needs further clarification.

The persistence of nightmares in migraine can dramatically reduce the quality of life of patients and generate or exacerbate anxiety, stress, or depression in affected individuals. In general, childhood nightmares can be persistent, so it is highly valuable to identify them in children with migraine and to apply treatment strategies to prevent this condition in adulthood. Therefore, it is important to include young adults and adolescents with migraine who are affected by nightmares in future studies. These studies might require special tools and strategies according to the needs of this population that must be considered in the study design. More studies are needed to shed light on precisely how the treatment of nightmares could help with the psychological symptoms and mood of patients with migraines. Mood and migraine are interrelated, and children are again the most affected group [116]. As emotion is related to dreams, further studies might also include migraine patients with complicated conditions such as grief or consider different cultural backgrounds and a diverse range of coping strategies.

Further studies are also encouraged to include other headache disorders (for example, post-traumatic headaches, considering that nightmares of PTSD are highly prevalent within the context of nightmare-related sleep disorders) in dream studies to establish a basis for diagnosis and comparison with migraine. In addition, larger cohorts and longitudinal studies can help determine how the pattern might be present at a time point and change over time in relation to age, sex, lifestyle, hormonal levels, cultural and behavioral coping strategies, occupation, etc. Comparing with normal participants without any headaches or nightmare disorders will also be beneficial for understanding the underlying reasons for this pattern.

Nightmares in migraine is an important field that still requires further investigation to provide evidence and determine the biological, psychological, or functional associations of nightmares and migraine, and optimal strategies to prevent and treat this co-occurring condition.

## Figures and Tables

**Table 1:** Proposed common neurotransmitters/neuromodulators in sleep and migraine (based on the literature [5,13,14,22,55,56]).

Neurotransmitter/Neuromodulator	Sleep	Migraine
Adenosine	NREM and REM sleep induction (A_1_ or A_2A_ receptor mediated)	Promotion of nociception (A_2A_ receptor mediated)
Dopamine	Consolidation of wakefulness	Promotion of antinociception (D_2_ receptor mediated)
Melatonin	Promotion of REM sleep, and promotion of NREM sleep in some conditions	Promotion of antinociception (MT_1_/MT_2_ receptor mediated)
Orexin	Promotion of wakefulness	Promotion of antinociception (OXR_1_ mediated)
Serotonin	Inhibition of REM and initiation of sleep	Promotion of antinociception (most likely via central serotonergic antinociceptive system. * Please note the dual role of serotonin in migraine.)
Others:		
GABA	Induction of deep NREM Stabilization of NREM sleep Reduction of REM sleep	Promotion of antinociception (most likely at the peripheral, spinal, and cortical levels. * Please note that the GABA_A_ and GABA_B_ receptors functionally complement each other, and each plays a role in control over trigemino thalamo cortical nociceptive transmission.)
Galanin	Promotion of NREM	Promotion of nociception at periphery, and antinociception centrally (* Please note the dual action.)
Histamine	Promotion of wakefulness	Promotion of nociception via H_1_; promotion of antinociception most likely via H_3_ (* Please note the dual action.)
Noradrenalin	Promotion of wakefulness	Promotion of nociception

REM: rapid eye movement sleep; NREM: non-rapid eye movement sleep; A: adenosine; D: dopamine; MT: melatonin; OXR: orexin receptor; GABA: gamma aminobutyric acid; H: histamine.

## References

[1] Cheatle M.D., Foster S., Pinkett A., Lesneski M., Qu D., Dhingra L. (2016). Assessing and Managing Sleep Disturbance in 466 Patients with Chronic Pain. Anesthesiol. Clin..

[2] Finan P.H., Goodin B.R., Smith M.T. (2013). The association of sleep and pain: An update and a path forward. J. Pain.

[3] Koffel E., Kroenke K., Bair M.J., Leverty D., Polusny M.A., Krebs E.E. (2016). The bidirectional relationship between sleep complaints and pain: Analysis of data from a randomized trial. Health Psychol..

[4] Andersen M.L., Araujo P., Frange C., Tufik S. (2018). Sleep Disturbance and Pain: A Tale of Two Common Problems. Chest.

[5] Haack M., Simpson N., Sethna N., Kaur S., Mullington J. (2020). Sleep deficiency and chronic pain: Potential underlying mechanisms and clinical implications. Neuropsychopharmacology.

[6] Tang N.K. (2008). Insomnia Co-Occurring with Chronic Pain: Clinical Features, Interaction, Assessments and Possible Interventions. Rev. Pain.

[7] Burgess H.J., Burns J.W., Buvanendran A., Gupta R., Chont M., Kennedy M., Bruehl S. (2019). Associations Between Sleep Disturbance and Chronic Pain Intensity and Function: A Test of Direct and Indirect Pathways. Clin. J. Pain.

[8] Korabelnikova E.A., Danilov A.B., Danilov A.B., Vorobyeva Y.D., Latysheva N.V., Artemenko A.R. (2020). Sleep Disorders and Headache: A Review of Correlation and Mutual Influence. Pain Ther..

[9] Sullivan D.P., Martin P.R., Boschen M.J. (2019). Psychological Sleep Interventions for Migraine and Tension-Type Headache: A Systematic Review and Meta-Analysis. Sci. Rep..

[10] Lund N., Westergaard M.L., Barloese M., Glumer C., Jensen R.H. (2014). Epidemiology of concurrent headache and sleep problems in Denmark. Cephalalgia.

[11] Vgontzas A., Pavlovic J.M. (2018). Sleep Disorders and Migraine: Review of Literature and Potential Pathophysiology Mechanisms. Headache.

[12] Ferini-Strambi L., Galbiati A., Combi R. (2019). Sleep disorder-related headaches. Neurol. Sci..

[13] Tiseo C., Vacca A., Felbush A., Filimonova T., Gai A., Glazyrina T., Hubalek I.A., Marchenko Y., Overeem L.H., Piroso S. (2020). Migraine and sleep disorders: A systematic review. J. Headache Pain.

[14] Waliszewska-Prosol M., Nowakowska-Kotas M., Chojdak-Lukasiewicz J., Budrewicz S. (2021). Migraine and Sleep-An Unexplained Association?. Int. J. Mol. Sci..

[15] Brennan K.C., Charles A. (2009). Sleep and headache. Semin. Neurol..

[16] Holland P.R. (2014). Headache and sleep: Shared pathophysiological mechanisms. Cephalalgia.

[17] Morgenthaler T.I., Auerbach S., Casey K.R., Kristo D., Maganti R., Ramar K., Zak R., Kartje R. (2018). Position Paper for the Treatment of Nightmare Disorder in Adults: An American Academy of Sleep Medicine Position Paper. J. Clin. Sleep Med..

[18] Patel D., Steinberg J., Patel P. (2018). Insomnia in the Elderly: A Review. J. Clin. Sleep Med..

[19] O’Hare M., Cowan R.P., Miglis M.G. (2017). Sleep and Headache. Sleep and Neurologic Disease.

[20] May A., Burstein R. (2019). Hypothalamic regulation of headache and migraine. Cephalalgia.

[21] Brown R.E., Basheer R., McKenna J.T., Strecker R.E., McCarley R.W. (2012). Control of sleep and wakefulness. Physiol. Rev..

[22] Fried N.T., Elliott M.B., Oshinsky M.L. (2017). The Role of Adenosine Signaling in Headache: A Review. Brain Sci..

[23] Liu Y.J., Chen J., Li X., Zhou X., Hu Y.M., Chu S.F., Peng Y., Chen N.H. (2019). Research progress on adenosine in central nervous system diseases. CNS Neurosci. Ther..

[24] Sheth S., Brito R., Mukherjea D., Rybak L.P., Ramkumar V. (2014). Adenosine receptors: Expression, function and regulation. Int. J. Mol. Sci..

[25] Vincenzi F., Pasquini S., Borea P.A., Varani K. (2020). Targeting Adenosine Receptors: A Potential Pharmacological Avenue for Acute and Chronic Pain. Int. J. Mol. Sci..

[26] Finan P.H., Smith M.T. (2013). The comorbidity of insomnia, chronic pain, and depression: Dopamine as a putative mechanism. Sleep Med. Rev..

[27] Aulinas A., Feingold K.R., Anawalt B., Boyce A., Chrousos G., de Herder W.W., Dhatariya K., Dungan K., Grossman A., Hershman J.M., Hofland J. (2000). Physiology of the Pineal Gland and Melatonin. Endotext.

[28] Brennan R., Jan J.E., Lyons C.J. (2007). Light, dark, and melatonin: Emerging evidence for the importance of melatonin in ocular physiology. Eye.

[29] Chen W.W., Zhang X., Huang W.J. (2016). Pain control by melatonin: Physiological and pharmacological effects. Exp. Ther. Med..

[30] Gelfand A.A., Goadsby P.J. (2016). The Role of Melatonin in the Treatment of Primary Headache Disorders. Headache.

[31] Long R., Zhu Y., Zhou S. (2019). Therapeutic role of melatonin in migraine prophylaxis: A systematic review. Medicine (Baltimore).

[32] Naegel S., Huhn J.I., Gaul C., Diener H.C., Obermann M., Holle D. (2017). No Pattern Alteration in Single Nocturnal Melatonin Secretion in Patients with Hypnic Headache: A Case-Control Study. Headache.

[33] Peres M.F. (2005). Melatonin, the pineal gland and their implications for headache disorders. Cephalalgia.

[34] Peres M.F., Masruha M.R., Zukerman E., Moreira-Filho C.A., Cavalheiro E.A. (2006). Potential therapeutic use of melatonin in migraine and other headache disorders. Expert Opin. Investig. Drugs.

[35] Song T.J., Kim B.S., Chu M.K. (2020). Therapeutic role of melatonin in migraine prophylaxis: Is there a link between sleep and migraine?. Prog. Brain Res..

[36] Wang C., Wang Q., Ji B., Pan Y., Xu C., Cheng B., Bai B., Chen J. (2018). The Orexin/Receptor System: Molecular Mechanism and Therapeutic Potential for Neurological Diseases. Front. Mol. Neurosci..

[37] Rains J.C., Poceta J.S. (2006). Headache and sleep disorders: Review and clinical implications for headache management. Headache.

[38] Alvaro P.K., Roberts R.M., Harris J.K. (2013). A Systematic Review Assessing Bidirectionality between Sleep Disturbances, Anxiety, and Depression. Sleep.

[39] Hamel E. (2007). Serotonin and migraine: Biology and clinical implications. Cephalalgia.

[40] Huang K.W., Ochandarena N.E., Philson A.C., Hyun M., Birnbaum J.E., Cicconet M., Sabatini B.L. (2019). Molecular and anatomical organization of the dorsal raphe nucleus. Elife.

[41] Jouvet M. (1999). Sleep and serotonin: An unfinished story. Neuropsychopharmacology.

[42] Monti J.M. (2010). The structure of the dorsal raphe nucleus and its relevance to the regulation of sleep and wakefulness. Sleep Med. Rev..

[43] Wang Q.P., Nakai Y. (1994). The dorsal raphe: An important nucleus in pain modulation. Brain Res. Bull..

[44] Engstrom M., Hagen K., Bjork M., Sand T. (2013). Answer to comment on “sleep quality, arousal and pain thresholds in migraineurs: A blinded controlled polysomnographic study”. J. Headache Pain.

[45] Engstrom M., Hagen K., Bjork M.H., Stovner L.J., Gravdahl G.B., Stjern M., Sand T. (2013). Sleep quality, arousal and pain thresholds in migraineurs: A blinded controlled polysomnographic study. J. Headache Pain.

[46] Vollono C., Testani E., Losurdo A., Mazza S., Della Marca G. (2013). Migraine, arousal and sleep deprivation: Comment on: “sleep quality, arousal and pain thresholds in migraineurs: A blinded controlled polysomnographic study”. J. Headache Pain.

[47] Ong J.C., Park M. (2012). Chronic headaches and insomnia: Working toward a biobehavioral model. Cephalalgia.

[48] Smith M.T., Haythornthwaite J.A. (2004). How do sleep disturbance and chronic pain inter-relate? Insights from the longitudinal and cognitive-behavioral clinical trials literature. Sleep Med. Rev..

[49] Spielman A.J., Caruso L.S., Glovinsky P.B. (1987). A behavioral perspective on insomnia treatment. Psychiatr. Clin. N. Am..

[50] Buenaver L.F., Quartana P.J., Grace E.G., Sarlani E., Simango M., Edwards R.R., Haythornthwaite J.A., Smith M.T. (2012). Evidence for indirect effects of pain catastrophizing on clinical pain among myofascial temporomandibular disorder participants: The mediating role of sleep disturbance. Pain.

[51] Tran D.P., Spierings E.L. (2013). Headache and insomnia: Their relation reviewed. Cranio.

[52] Odegard S.S., Engstrom M., Sand T., Stovner L.J., Zwart J.A., Hagen K. (2010). Associations between sleep disturbance and primary headaches: The third Nord-Trondelag Health Study. J. Headache Pain.

[53] Rains J.C. (2018). Sleep and Migraine: Assessment and Treatment of Comorbid Sleep Disorders. Headache.

[54] Goadsby P.J., Holland P.R., Martins-Oliveira M., Hoffmann J., Schankin C., Akerman S. (2017). Pathophysiology of Migraine: A Disorder of Sensory Processing. Physiol. Rev..

[55] Sokolov A.Y., Lyubashina O.A., Amelin A.V., Panteleev S.S. (2014). The role of gamma-aminobutyric acid in migraine pathogenesis. Neurochem. J..

[56] Kilinc E., Guerrero-Toro C., Zakharov A., Vitale C., Gubert-Olive M., Koroleva K., Timonina A., Luz L.L., Shelukhina I., Giniatullina R. (2017). Serotonergic mechanisms of trigeminal meningeal nociception: Implications for migraine pain. Neuropharmacology.

[57] Pilcher J.J., Ginter D.R., Sadowsky B. (1997). Sleep quality versus sleep quantity: Relationships between sleep and measures of health, well-being and sleepiness in college students. J. Psychosom. Res..

[58] Bruni O., Russo P.M., Violani C., Guidetti V. (2004). Sleep and migraine: An actigraphic study. Cephalalgia.

[59] Nayak C., Sinha S., Nagappa M., Nagaraj K., Kulkarni G.B., Thennarasu K., Taly A.B. (2016). Study of sleep microstructure in patients of migraine without aura. Sleep Breath.

[60] Lin Y.K., Lin G.Y., Lee J.T., Lee M.S., Tsai C.K., Hsu Y.W., Lin Y.Z., Tsai Y.C., Yang F.C. (2016). Associations Between Sleep Quality and Migraine Frequency: A Cross-Sectional Case-Control Study. Medicine (Baltimore).

[61] Zhu Z., Fan X., Li X., Tan G., Chen L., Zhou J. (2013). Prevalence and predictive factors for poor sleep quality among migraineurs in a tertiary hospital headache clinic. Acta Neurol. Belg..

[62] Sateia M.J. (2014). International classification of sleep disorders-third edition: Highlights and modifications. Chest.

[63] Li S.X., Zhang B., Li A.M., Wing Y.K. (2010). Prevalence and correlates of frequent nightmares: A community-based 2-phase study. Sleep.

[64] Rek S., Sheaves B., Freeman D. (2017). Nightmares in the general population: Identifying potential causal factors. Soc. Psychiatry Psychiatr. Epidemiol..

[65] Woodward S.H., Arsenault N.J., Murray C., Bliwise D.L. (2000). Laboratory sleep correlates of nightmare complaint in PTSD inpatients. Biol. Psychiatry.

[66] Semiz U.B., Basoglu C., Ebrinc S., Cetin M. (2008). Nightmare disorder, dream anxiety, and subjective sleep quality in patients with borderline personality disorder. Psychiatry Clin. Neurosci..

[67] Sheaves B., Onwumere J., Keen N., Stahl D., Kuipers E. (2015). Nightmares in Patients with Psychosis: The Relation With Sleep, Psychotic, Affective, and Cognitive Symptoms. Can. J. Psychiatry.

[68] Gieselmann A., Ait Aoudia M., Carr M., Germain A., Gorzka R., Holzinger B., Kleim B., Krakow B., Kunze A.E., Lancee J. (2019). Aetiology and treatment of nightmare disorder: State of the art and future perspectives. J. Sleep Res..

[69] Pigeon W.R., Pinquart M., Conner K. (2012). Meta-analysis of sleep disturbance and suicidal thoughts and behaviors. J. Clin. Psychiatry.

[70] Phelps A.J., Forbes D., Creamer M. (2008). Understanding posttraumatic nightmares: An empirical and conceptual review. Clin. Psychol. Rev..

[71] Robert G., Zadra A. (2014). Thematic and content analysis of idiopathic nightmares and bad dreams. Sleep.

[72] Jenkins D. (2019). Nightmare resolution: Where to begin, where to end? A commentary on “The mechanisms of action underlying the efficacy of psychological nightmare treatments: A systematic review and thematic analysis of discussed hypotheses”. Sleep Med. Rev..

[73] Rousseau A., Belleville G. (2018). The mechanisms of action underlying the efficacy of psychological nightmare treatments: A systematic review and thematic analysis of discussed hypotheses. Sleep Med. Rev..

[74] Vgontzas A., Cui L., Merikangas K.R. (2008). Are sleep difficulties associated with migraine attributable to anxiety and depression?. Headache.

[75] Levitan H. (1984). Dreams which culminate in migraine headaches. Psychother. Psychosom..

[76] Lippman C.W. (1954). Recurrent dreams in migraine: An aid to diagnosis. J. Nerv. Ment. Dis..

[77] Heather-Greener G.Q., Comstock D., Joyce R. (1996). An investigation of the manifest dream content associated with migraine headaches: A study of the dreams that precede nocturnal migraines. Psychother. Psychosom..

[78] Gallego-Mere A. (1989). The manifest content of dreams. Am. J. Psychoanal..

[79] Cartwright R.D. (1979). The nature and function of repetitive dreams: A survey and speculation. Psychiatry.

[80] Kales A., Soldatos C.R., Caldwell A.B., Charney D.S., Kales J.D., Markel D., Cadieux R. (1980). Nightmares: Clinical characteristics and personality patterns. Am. J. Psychiatry.

[81] Wright J., Koulack D. (1987). Dreams and contemporary stress: A disruption-avoidance-adaptation model. Sleep.

[82] Solms M. (2000). Dreaming and REM sleep are controlled by different brain mechanisms. Behav. Brain Sci..

[83] Domhoff G.W. (1996). The Hall/Van de Castle System of Content Analysis. Finding Meaning in Dreams. Emotions, Personality, and Psychotherapy.

[84] Jonckheere P. (1971). The chronic headache patient. A psychodynamic study of 30 cases, compared with cardio-vascular patients. Psychother. Psychosom..

[85] Rees W.L. (1974). A controlled epidemiological study of the role of psychological factors in migraine. Arch. Neurobiol. (Madr.).

[86] Greenberg R.P., O’Neill R.M. (1988). The construct validity of the MMPI alexithymia scale with psychiatric inpatients. J. Pers. Assess..

[87] Pulver S.E., Renik O. (1984). The clinical use of the manifest dream. Panel report. J. Am. Psychoanal Assoc..

[88] Biondi M., Portuesi G. (1994). Tension-type headache: Psychosomatic clinical assessment and treatment. Psychother. Psychosom..

[89] Gottschalk L.A. (1995). Uses of dreams. Psychother. Psychosom..

[90] Foral P., Knezevich J., Dewan N., Malesker M. (2011). Medication-induced sleep disturbances. Consult. Pharm..

[91] Ghaffarinejad A., Mehdizadeh Zareanari A., Pouya F. (2015). Investigating the Dreaming Content of Migraineurs. Zahedan J. Res. Med. Sci..

[92] De Angeli F., Lovati C., Giani L., Mariotti D’Alessandro C., Raimondi E., Scaglione V., Castoldi D., Capiluppi E., Mariani C. (2014). Negative emotions in migraineurs dreams: The increased prevalence of oneiric fear and anguish, unrelated to 660 mood disorders. Behav. Neurol..

[93] Lovati C., DeAngeli F., D’Amico D., Giani L., D’Alessandro C.M., Zardoni M., Scaglione V., Castoldi D., Capiluppi E., Curone M. (2014). Is the brain of migraineurs “different” even in dreams?. Neurol. Sci..

[94] Suzuki K., Miyamoto T., Miyamoto M., Suzuki S., Watanabe Y., Takashima R., Hirata K. (2013). Dream-enacting behaviour is associated with impaired sleep and severe headache-related disability in migraine patients. Cephalalgia.

[95] Nadorff M.R., Lambdin K.K., Germain A. (2014). Pharmacological and non-pharmacological treatments for nightmare disorder. Int. Rev. Psychiatry.

[96] Selvanathan J., Pham C., Nagappa M., Peng P.W.H., Englesakis M., Espie C.A., Morin C.M., Chung F. (2021). Cognitive behavioral therapy for insomnia in patients with chronic pain - A systematic review and meta-analysis of randomized controlled trials. Sleep Med. Rev..

[97] Harris P., Loveman E., Clegg A., Easton S., Berry N. (2015). Systematic review of cognitive behavioural therapy for the management of headaches and migraines in adults. Br. J. Pain.

[98] Smitherman T.A., Kuka A.J., Calhoun A.H., Walters A.B.P., Davis-Martin R.E., Ambrose C.E., Rains J.C., Houle T.T. (2018). Cognitive-Behavioral Therapy for Insomnia to Reduce Chronic Migraine: A Sequential Bayesian Analysis. Headache.

[99] Crawford M.R., Luik A.I., Espie C.A., Taylor H.L., Burgess H.J., Jones A.L., Ong J.C., Team R.U.S.R. (2020). Digital Cognitive Behavioral Therapy for Insomnia in Women With Chronic Migraines. Headache.

[100] Martin P.R., Reece J., Callan M., MacLeod C., Kaur A., Gregg K., Goadsby P.J. (2014). Behavioral management of the triggers of recurrent headache: A randomized controlled trial. Behav. Res. Ther..

[101] Ree M., Junge M., Cunnington D. (2017). Australasian Sleep Association position statement regarding the use of psychological/behavioral treatments in the management of insomnia in adults. Sleep Med..

[102] Cervena K., Dauvilliers Y., Espa F., Touchon J., Matousek M., Billiard M., Besset A. (2004). Effect of cognitive behavioural therapy for insomnia on sleep architecture and sleep EEG power spectra in psychophysiological insomnia. J. Sleep Res..

[103] Rains J.C. (2008). Chronic headache and potentially modifiable risk factors: Screening and behavioral management of sleep disorders. Headache.

[104] Yang C.P., Wang S.J. (2017). Sleep in Patients with Chronic Migraine. Curr. Pain Headache Rep..

[105] Lee Y.G., Kim S., Kim N., Choi J.W., Park J., Kim S.J., Gwak A.R., Lee Y.J. (2018). Changes in subcortical resting-state functional connectivity in patients with psychophysiological insomnia after cognitive-behavioral therapy: Changes in resting-state FC after CBT for insomnia patients. Neuroimage Clin..

[106] Smith M.T., Perlis M.L., Chengazi V.U., Soeffing J., McCann U. (2005). NREM sleep cerebral blood flow before and after behavior therapy for chronic primary insomnia: Preliminary single photon emission computed tomography (SPECT) data. Sleep Med..

[107] Spielman A.J., Saskin P., Thorpy M.J. (1987). Treatment of chronic insomnia by restriction of time in bed. Sleep.

[108] Zappaterra M., Jim L., Pangarkar S. (2014). Chronic pain resolution after a lucid dream: A case for neural plasticity?. Med. Hypotheses.

[109] van Schagen A., Lancee J., Swart M., Spoormaker V., van den Bout J. (2017). Nightmare Disorder, Psychopathology Levels, and Coping in a Diverse Psychiatric Sample. J. Clin. Psychol..

[110] Robblee J., Starling A.J. (2019). SEEDS for success: Lifestyle management in migraine. Cleve Clin. J. Med..

[111] Merrill R.M., Aldana S.G., Greenlaw R.L., Diehl H.A., Salberg A. (2007). The effects of an intensive lifestyle modification program on sleep and stress disorders. J. Nutr. Health Aging.

[112] Gazerani P. (2021). A Bidirectional View of Migraine and Diet Relationship. Neuropsychiatr. Dis. Treat..

[113] Gazerani P. (2020). Migraine and Diet. Nutrients.

[114] Amin F.M., Aristeidou S., Baraldi C., Czapinska-Ciepiela E.K., Ariadni D.D., Di Lenola D., Fenech C., Kampouris K., Karagiorgis G., Braschinsky M. (2018). The association between migraine and physical exercise. J. Headache Pain.

[115] Firth J., Solmi M., Wootton R.E., Vancampfort D., Schuch F.B., Hoare E., Gilbody S., Torous J., Teasdale S.B., Jackson S.E. (2020). A meta-review of “lifestyle psychiatry”: The role of exercise, smoking, diet and sleep in the prevention and treatment of mental disorders. World Psychiatry.

[116] Gazerani P. (2021). Migraine and Mood in Children. Behav. Sci..

